# Supplementary material for: An exploration of the professional identity of clinical academics using repertory grid technique
Source: PLoS One. 2022 Nov 17;17(11):e0277361. doi: 10.1371/journal.pone.0277361 (PMC9671447; doi:10.1371/journal.pone.0277361)
Supplement: S1 File — (DOCX) [file pone.0277361.s001.docx]

# S1 File: % Similarity Score Calculation

To begin with, a sum of differences (SD) for each element was calculated from the ratings for each construct by subtracting the smaller number from the larger number along the rows and summing the total. To enable comparison across multiple grids, the SD is converted to a percentage similarity score using the following formula where LR= largest rating (in this case, 5 because a 5-point scale was used) and C= number of constructs in the grid:

$$\% similarity score=100-\left\{ \frac{SD}{\left[ \left( LR-1 \right)x C \right]} \right\}x100$$

The higher the % similarity score, the more similar two elements are.

A similar process is followed to calculate the % similarity score for constructs, with one significant difference. Because constructs are bipolar, they have the same meaning when the ratings are reversed, and since triads are randomly selected, the designation of the emerging poles and implicit poles is arbitrary. Therefore, to ensure no relationships are missed, constructs are compared to each other twice: firstly, when the ratings are as given, and secondly when the ratings of one construct are reversed. When ratings are reversed, an element with a score of 1 becomes 5, a score of 2 becomes 4 and so on. The highest rating of the two is taken as the % similarity score. The formula for calculating % similarity scores of constructs is slightly different because the range of possible percentages is spread over a 200-point scale, rather than a 100-point scale:

$$\% similarity score=100-\left\{ \frac{SD}{\left[ \left( LR-1 \right)xE \right]} \right\}x200$$

Where E= the number of elements (Jankowicz, 2004).

JANKOWICZ, D. 2004. *The easy guide to repertory grids,* University of Luton, UK, Wiley.
