# Supplementary material for: An exploration of the professional identity of clinical academics using repertory grid technique
Source: PLoS One. 2022 Nov 17;17(11):e0277361. doi: 10.1371/journal.pone.0277361 (PMC9671447; doi:10.1371/journal.pone.0277361)
Supplement: S2 File — (DOCX) [file pone.0277361.s002.docx]

# S2 File: Core categorisation procedure

| **Overarching construct** | **Codes** | **Frequency** | **Total frequency** |
| --- | --- | --- | --- |
| Willing to help others, altruistic vs focussed on own goals | Good mentor, interested in mentorship vs poor mentor, not interested in mentorship | 7 | 20 |
|  | Committed to teaching vs not committed to teaching | 6 |  |
|  | Willing to help others vs focussed on own goals | 3 |  |
|  | Helps develop research infrastructure for all vs focussed on own research infrastructure | 2 |  |
|  | Engages with own institutional admin vs doesn’t engage with own institutional admin | 2 |  |
| Research outputs have significant impact vs outputs have lower impact | Significant contribution to discipline vs less significant contribution to discipline | 8 | 13 |
|  | Research with international significance vs research without international significance | 2 |  |
|  | Leading change in practice through research vs not effecting change | 1 |  |
|  | Influencing public policy vs not engaging with public policy | 1 |  |
|  | Collaboration with industry, develops own IP vs no collaboration with industry | 1 |  |
| Agreeable, approachable vs antagonistic, intimidating | Approachable vs intimidating, brusque | 8 | 12 |
|  | Supports colleagues’ work vs excessively critical | 1 |  |
|  | Younger, more relatable vs older, less relatable | 1 |  |
|  | Supportive supervisor vs not a supportive supervisor | 1 |  |
|  | Respects work-life boundaries of colleagues vs doesn’t respect work-life boundaries | 1 |  |
| Works to build a network of collaborators vs prefers to work alone | Good at collaborating vs not good at collaborating | 5 | 11 |
|  | Interested in collaborating vs inward-looking, not collaborative | 5 |  |
|  | International work experience vs no international work experience | 1 |  |
| Access to resources vs less access to resources | Poorly resourced research environment vs well-resourced research environment | 3 | 9 |
|  | Head of discipline/well-established, more access to resources vs does not head discipline/not well established, less access to resources | 2 |  |
|  | Has protected academic time vs no protected academic time | 2 |  |
|  | Small research team vs large research team | 1 |  |
|  | Ability to acquire research funding vs not focussed on research funding | 1 |  |
| Focus on clinical work vs focus on research | Focus on patient care vs focus on research | 5 | 9 |
|  | Active clinician, excellent clinical skills vs less active clinically, average clinical skills | 4 |  |
| Committed to public outreach vs not committed to public outreach |  |  | 7 |
| Established researcher, well-recognised in scientific community vs not well recognised as a researcher | International expert, niche area vs national expert, broad area | 3 | 7 |
|  | Sought-after speaker vs not sought-after | 2 |  |
|  | Well-established vs less well recognised | 1 |  |
|  | Engage with organisations in their field vs doesn’t engage with organisations in their field | 1 |  |
| Innovative, embracing new ideas vs closed-minded, lacking imagination | Innovative vs not innovative | 3 | 6 |
|  | Inclusive and open-minded vs dismissive and closed-minded | 1 |  |
|  | Open to new technology vs traditional approach | 1 |  |
|  | Interdisciplinary collaborations, exchange of ideas vs no interdisciplinary collaborations | 1 |  |
| Honesty and integrity vs self-serving | Scientific rigor vs less rigorous approach | 1 | 5 |
|  | Research integrity vs unreliability as a researcher | 1 |  |
|  | Provides accurate information to the public vs provides inaccurate information to the public | 1 |  |
|  | Integrity vs self-serving | 1 |  |
|  | Can acquire peer-reviewed funding vs reliance on soft funding | 1 |  |
| Excellent, inspiring teacher vs poor, boring teacher | Good at teaching vs not good at teaching | 4 | 5 |
|  | Inspirational teacher vs boring teacher | 1 |  |
| Experienced researcher vs early career, less experienced | Older age, more experience vs younger, less experience | 2 | 5 |
|  | Late career, less interest in new projects vs mid-career, more interest in new projects | 3 |  |
| Greater demands on time outside work vs fewer demands on time outside work | Female vs male | 3 | 4 |
|  | Greater childcare responsibilities vs fewer childcare responsibilities | 1 |  |
| Democratic leader, fosters autonomy vs autocratic, micromanager | Leadership which fosters autonomy vs micromanager | 2 | 4 |
|  | Democratic leadership style vs autocratic leadership style | 1 |  |
|  | Motivational leadership vs lowers morale | 1 |  |
| Dedicated and hardworking vs lazy, inefficient | Hardworking vs lazy | 1 | 4 |
|  | Dedicated vs not dedicated | 1 |  |
|  | Time efficient vs not time efficient | 1 |  |
|  | Excels in clinical, research and teaching vs one-dimensional | 1 |  |
| Pure clinical research vs basic scientific research |  |  | 3 |
| Synergy between clinical and research work vs disconnect between clinical and research work |  |  | 3 |
| Good at communicating vs not good at communicating |  |  | 2 |
| Working excessive hours vs good work-life balance |  |  | 2 |
| Surgeon vs physician |  |  | 2 |
