# Supplementary material for: An exploration of the professional identity of clinical academics using repertory grid technique
Source: PLoS One. 2022 Nov 17;17(11):e0277361. doi: 10.1371/journal.pone.0277361 (PMC9671447; doi:10.1371/journal.pone.0277361)
Supplement: S3 File — (DOCX) [file pone.0277361.s003.docx]

# S3 File: Differential Analysis

**Table 1: Established vs. trainee clinical academics**

| **Overarching construct** | **Frequency Established (%)** | **Frequency Trainee (%)** | **Importance Established** | **Importance Trainee** |
| --- | --- | --- | --- | --- |
| Willing to help others, altruistic vs focussed on own goals | 13 (19.1) | 7 (10.8) | Intermediate | High |
| Research outputs have significant impact vs outputs have lower impact | 6 (8.8) | 7 (10.8) | High | High |
| Agreeable, approachable vs antagonistic, intimidating | 5 (7.4) | 7 (10.8) | Intermediate | Low |
| Works to build a network of collaborators vs prefers to work alone | 6 (8.8) | 5 (7.7) | High | High |
| Access to resources vs less access to resources | 2 (2.9) | 7 (10.8) | Intermediate | Intermediate |
| Focus on clinical work vs focus on research | 5 (7.4) | 4 (6.2) | No consensus | Intermediate |
| Committed to public outreach vs not committed to public outreach | 3 (4.4) | 4 (6.2) | High | Intermediate |
| Established researcher, well-recognised in scientific community vs not well recognised as a researcher | 3 (4.4) | 4 (6.2) | High | High |
| Innovative, embracing new ideas vs closed-minded, lacking imagination | 2 (2.9) | 4 (6.2) | High | Intermediate |
| Honesty and integrity vs self-serving | 5 (7.4) | 0 (0) | High | N/A |
| Excellent, inspiring teacher vs poor, boring teacher | 3 (4.4) | 2 (3.1) | High | High |
| Experienced researcher vs early career, less experienced | 3 (4.4) | 2 (3.1) | Low | No consensus |
| Greater demands on time outside work vs fewer demands on time outside work | 3 (4.4) | 1 (1.5) | Low | Low |
| Democratic leader, fosters autonomy vs autocratic, micromanager | 1 (1.5) | 3 (4.6) | Intermediate | Low |
| Dedicated and hardworking vs lazy, inefficient | 3 (4.4) | 1 (1.5) | Intermediate | Intermediate |
| Pure clinical research vs basic scientific research | 2 (2.9) | 1 (1.5) | No consensus | Intermediate |
| Synergy between clinical and research work vs disconnect between clinical and research work | 0 (0) | 3 (4.6) | Not Applicable (N.A.) | Intermediate |
| Good at communicating vs not good at communicating | 1 (1.5) | 1 (1.5) | Intermediate | Intermediate |
| Working excessive hours vs good work-life balance | 1 (1.5) | 1 (1.5) | Low | High |
| Surgeon vs physician | 1 (1.5) | 1 (1.5) | Low | Low |

**Table 2: Female vs. male clinical academics**

| **Overarching construct** | **Frequency female (%)** | **Frequency male (%)** | **Importance female** | **Importance male** |
| --- | --- | --- | --- | --- |
| Willing to help others, altruistic vs focussed on own goals | 8 (11.8) | 10 (15.4) | Intermediate | Intermediate |
| Research outputs have significant impact vs outputs have lower impact | 6 (8.8) | 7 (10.7) | High | High |
| Agreeable, approachable vs antagonistic, intimidating | 8 (11.8) | 4 (6.2) | Intermediate | High |
| Works to build a network of collaborators vs prefers to work alone | 7 (10.3) | 4 (6.2) | High | High |
| Access to resources vs less access to resources | 5 (7.4) | 4 (6.2) | Intermediate | Intermediate |
| Focus on clinical work vs focus on research | 5 (7.4) | 4 (6.2) | High | No consensus |
| Committed to public outreach vs not committed to public outreach | 5 (7.4) | 2 (3) | Intermediate | No consensus |
| Established researcher, well-recognised in scientific community vs not well recognised as a researcher | 1 (1.5) | 6 (9.2) | High | High |
| Innovative, embracing new ideas vs closed-minded, lacking imagination | 1 (1.5) | 5 (7.7) | Intermediate | High |
| Honesty and integrity vs self-serving | 2 (2.9) | 3 (4.6) | High | High |
| Excellent, inspiring teacher vs poor, boring teacher | 3 (4.4) | 2 (3) | High | High |
| Experienced researcher vs early career, less experienced | 2 (2.9) | 3 (4.6) | Low | Intermediate |
| Greater demands on time outside work vs fewer demands on time outside work | 4 (5.9) | 0 (0) | Low | N/A |
| Democratic leader, fosters autonomy vs autocratic, micromanager | 2 (2.9) | 2 (3) | No consensus | No consensus |
| Dedicated and hardworking vs lazy, inefficient | 1 (1.5) | 3 (4.6) | Intermediate | Intermediate |
| Pure clinical research vs basic scientific research | 3 (4.4) | 2 (3) | Low | Intermediate |
| Synergy between clinical and research work vs disconnect between clinical and research work | 2 (2.9) | 1 (1.5) | No consensus | Intermediate |
| Good at communicating vs not good at communicating | 0 (0) | 2 (3) | N/A | Intermediate |
| Working excessive hours vs good work-life balance | 1 (1.5) | 1 (1.5) | Low | High |
| Surgeon vs physician | 2 (2.9) | 0 (0) | Low | N/A |
