# Supplementary material for: An exploration of the professional identity of clinical academics using repertory grid technique
Source: PLoS One. 2022 Nov 17;17(11):e0277361. doi: 10.1371/journal.pone.0277361 (PMC9671447; doi:10.1371/journal.pone.0277361)
Supplement: Attachment — Submitted filename: Response to reviewers.docx [file pone.0277361.s004.docx]

# Author’s response to Reviewers

## PONE-D-22-16880

## An exploration of the professional identity of clinical academics using repertory grid technique

## PLOS ONE

We wish to take the opportunity to thank the Editor and Reviewers 1 and 2 for their very helpful and insightful comments. We have addressed the comments below and include references to the exact location within the full manuscript.

| Reviewer/Editor Comments | Authors’ Response | Amendment with Exact location in full manuscript (no track changes), (Page(s), line number(s) |
| --- | --- | --- |
| Please ensure that your manuscript meets PLOS ONE's style requirements, including those for file naming | I have reviewed the style requirements and amended the manuscript accordingly | Page 1, line 8: corresponding author’s initials added after email address  Page 8, line 151: table caption moved above table  Page 10, line 195: figure caption added to end of paragraph  Page 10, line 202: table caption moved above table  Page 14, line 280-281: table caption moved above table |
| " Unfunded study"  At this time, please address the following queries:  a)        Please clarify the sources of funding (financial or material support) for your study. List the grants or organizations that supported your study, including funding received from your institution.  b)        State what role the funders took in the study. If the funders had no role in your study, please state: “The funders had no role in study design, data collection and analysis, decision to publish, or preparation of the manuscript.”  c)        If any authors received a salary from any of your funders, please state which authors and which funders.  d)        If you did not receive any funding for this study, please state: “The authors received no specific funding for this work.”  Please include your amended statements within your cover letter; we will change the online submission form on your behalf. | The authors received no specific funding for this work | The amended statement is included in the cover letter |
| In your Data Availability statement, you have not specified where the minimal data set underlying the results described in your manuscript can be found. PLOS defines a study's minimal data set as the underlying data used to reach the conclusions drawn in the manuscript and any additional data required to replicate the reported study findings in their entirety. All PLOS journals require that the minimal data set be made fully available. For more information about our data policy, please see <http://journals.plos.org/plosone/s/data-availability>. | The minimal data set has been included in the supplementary information. This indicates the core categorisation procedure: readers will be able to see the codes that formed the overarching constructs and the frequency with which they arose. | Page 26, line 599: Supporting information  **S2 File: Core categorisation procedure**  This file is cited on Pg 13, line 267  S2 file submitted with manuscript |
| Please include captions for your Supporting Information files at the end of your manuscript, and update any in-text citations to match accordingly. Please see our Supporting Information guidelines for more information: <http://journals.plos.org/plosone/s/supporting-information>. | Caption and in text citations added as described above |  |
| Please review your reference list to ensure that it is complete and correct. If you have cited papers that have been retracted, please include the rationale for doing so in the manuscript text, or remove these references and replace them with relevant current references. Any changes to the reference list should be mentioned in the rebuttal letter that accompanies your revised manuscript. If you need to cite a retracted article, indicate the article’s retracted status in the References list and also include a citation and full reference for the retraction notice. | I have reviewed the reference list and can confirm that it is complete and correct. Links to all papers have been checked and as of 12^th^ October 2022, none have been retracted. |  |
| The introduction needs to be more in-depth, emphasizing the problem; the importance of the study is unclear. | I have rewritten the opening paragraph to emphasise the problem and the importance of the study | Pg 4, Lines 47-57  Increases in clinical research funding over the past number of decades have not seen a parallel increase in cures and treatments for human disease, and the inability to translate basic scientific findings into treatments, combined with other inefficiencies, results in a wastage of up to 85% of research funding, estimated to cost $200 billion annually (1, 2). Clinical academics, or clinician scientists combine clinical practice with research, are well-positioned to offer critical insights and identify pertinent research questions, helping to bridge the gap between research and patient care. They have contributed to some of the most fundamental breakthroughs in medicine in recent history (3) and research led by MDs is twice as likely to involve humans as PhD-led research (4). However, despite the importance of the role, the clinical academic workforce is under threat, and there have been international calls to build and sustain it for the future (5-8). |
| Please bring more elements that give more clarity to the study rationale. | The rationale of the study is further explained in the second and fourth paragraphs of the introduction | Pg 4, lines 62-66  Current definitions of the clinician scientist role tend to focus exclusively on research funding obtained, a superficial definition which does not acknowledge the many facets of this complex role (11). Maintaining and supporting the clinician academic workforce requires a deeper understanding of the abilities and attributes of clinical academics.  Pg 5, lines 83-88  Describing the personal and environmental factors that support a successful clinical academic career will contribute to the understanding of the professional identity of clinical academics, which may be a critical determinant of career stability ; further, a greater understanding of the professional identity of clinical academics may inform selection criteria for recruitment and promotion and the development of education and training strategies to support the clinical academics of the future (16). |
| More than 60% of the references used in the text were published more than 5 years ago. Please update. | I have repeated a literature review and included recent studies of relevance, e.g.:  Eshel et al, 2022; Bensken et al, 2019; Lent et al, 2019; Byram et al, 2022 |  |
| Considering that the term professional identity is a rather broad concept and is structured differently depending on the theoretical foundation on which it is based, I think that the text would benefit from a more precise allusion to the concept, from the point of view of the theory that the authors adopt. The text implies that the professional identity of physician-investigators is imbued with personal characteristics and values, developed in interaction with organizational aspects, but the concept is not explicitly outlined. In this direction, it is necessary to consider that the professional identity is more than the sum of the characteristics of success and this could be better explored. | This paper explores the professional identity of the clinical academic which is made up of characteristics, values, and norms which are internalised over time. I have clarified what I mean by the term “professional identity”, as you say it is a broad term and can have different meanings depending on the context. Exploring the characteristics of a successful clinical academic can contribute to the understanding of the professional identity of clinical academics, but only forms part of the picture. I have expanded one paragraph of the introduction, and used phrases such as “contribute to the understanding” to emphasise that characteristics alone do not form a complete understanding of professional identity.  Social Cognitive Career Theory has recently been used to explore professional identity formation in clinician educators (Byram et al 2022), and a reference to this study has been added. | Page5, lines 745-88  Professional identity in physicians has been defined as a set of characteristics, values and norms which become internalised over time; eventually the individual thinks, acts and feels like a physician (15). Understanding professional identity and how it is formed requires an exploration of key attributes and determinants of success or failure (15, 16), although this only forms part of the picture. Competency frameworks have been developed which show a more rounded view of the work of clinical academics (17), however, there remains a gap in the knowledge on how clinical academics develop a professional identity which integrates the dual disciplines of research and clinical practice, and what the critical determinants of success or failure are (16). Describing the personal and environmental factors that support a successful clinical academic career will contribute to the understanding of the professional identity of clinical academics, which may be a critical determinant of career stability ; further, a greater understanding of the professional identity of clinical academics may inform selection criteria for recruitment and promotion and the development of education and training strategies to support the clinical academics of the future (16).  Page 6, line 102-105  It has also been shown to provide an effective theoretical foundation for exploring clinical academic and clinician educator career development and professional identity formation (21-23). |
| With regard to the qualitative aspect of data collection and analysis, I suggest that some points be clarified according to the COREq guideline: 1. Were only the interviewer and the interviewee present at the time of the virtual interview?  2. Which virtual platform was used for the interviews?  3. What precautions were established to ensure a safe environment for verbalizing aspects related to the career?  4. Were there refusals to participate in the study? What were the reasons?  5. Is there any characterization of the participants, in addition to gender, that deserves to be mentioned?  6. How long did the interviews last | I have clarified the points raised in the adjacent column.  In response to the fifth point raised, have now included the differential analyses we carried out between established and trainee clinicians, and female and male clinicians. This was initially omitted in the interest of brevity, however there were some interesting findings, so we have now included a summary of this analysis. Information has been added to the methods, results and discussion sections. The tables pertaining to this analysis are quite large so I chose to include them separately in a supplemental file. | 1. Page 8, lines 159-160  only the interviewer and interviewee were present  2. Page 8, lines 156-157  Ten interviews were conducted virtually using downloadable computer software for video communications, ZOOM®,  3. Page 8, lines 155-156  Informed written consent was obtained from all participants.  Page 9, lines 173-177  Participants did not disclose the names of the elements to the interviewer, they assigned elements a letter A-H and referred to them only by their assigned letter. The interviewer therefore was not aware of the identities of the elements. This permitted a more open discussion and avoided concerns about identification of elements in a potentially negative way.  4. Page 8, lines 147-149  Two of the clinical academics we invited declined to participate; one because they felt they were too junior to be able to discuss the topic in detail, and the other because of time constraints.  5. Multiple additions:  Page 2, line 30-31  A differential analysis between established/trainee and female/male participants was carried out.  Page 3, line 38-40  Female participants had greater concerns about the impact of familial responsibilities on career progression.  Page 13, lines 258-262 (paragraph under heading “Differential analysis”)  Page 15, lines 285-298 (paragraph under heading “Differential analysis findings”)  Page 20, lines 411-439 (paragraph under heading “Comparison of established and trainee, female and male clinical academics”)  Tables to illustrate the findings from the differential analysis are included in a supplemental file, S3 File  6. Page 8, line 160  Interviews lasted between 49 minutes and 1 hour 22 minutes. |
| I consider it important that the authors point out that the elements of success versus insucess mentioned refer to a given well-defined socio-cultural location, this will naturally differ in other sociocultural realities of medical education (for example, developing countries). A note in this sense, in the discussion, would be very relevant, since the identified constructs concern a given historical and cultural reality. | We agree this is an important point, and is now included in the discussion under the Limitations heading. | Page 21, Lines 449-452: Participants were not geographically dispersed although they represented two different healthcare systems. Views from clinical academics in other sociocultural locations are therefore absent which creates a limitation to our understanding of the clinical academic in other locations. |
